# A Review of Bandgap Engineering and Prediction in 2D Material Heterostructures: A DFT Perspective

**DOI:** 10.3390/ijms252313104

**Published:** 2024-12-06

**Authors:** Yoonju Oh, Seunghyun Song, Joonho Bae

**Affiliations:** Department of Physics, Gachon University, Seongnum-si 13120, Gyeonggi-do, Republic of Korea; oyj0413@gmail.com (Y.O.); songsh139@gmail.com (S.S.)

**Keywords:** density functional theory, heterostructures, bandgap engineering, first-principles study

## Abstract

The advent of two-dimensional (2D) materials and their capacity to form van der Waals (vdW) heterostructures has revolutionized numerous scientific fields, including electronics, optoelectronics, and energy storage. This paper presents a comprehensive investigation of bandgap engineering and band structure prediction in 2D vdW heterostructures utilizing density functional theory (DFT). By combining various 2D materials, such as graphene, hexagonal boron nitride (h-BN), transition metal dichalcogenides, and blue phosphorus, these heterostructures exhibit tailored properties that surpass those of individual components. Bandgap engineering represents an effective approach to addressing the limitations inherent in material properties, thereby providing enhanced functionalities for a range of applications, including transistors, photodetectors, and solar cells. Furthermore, this study discusses the current limitations and challenges associated with bandgap engineering in 2D heterostructures and highlights future prospects aimed at unlocking their full potential for advanced technological applications.

## 1. Introduction

The advent of heterostructures composed of two-dimensional (2D) materials has transformed various scientific fields, including electronics, optoelectronics, and energy storage. By combining layers of different 2D materials, these heterostructures exhibit tailored properties that surpass those of individual materials, opening new possibilities for advanced device applications and efficient energy solutions. Heterostructures are composite materials made by stacking different layers of semiconductor materials such as graphene, hexagonal boron nitride (h-BN), transition metal dichalcogenides (TMDs) such as MoS_2_ and WSe_2_, and black phosphorus [1]. Van der Waals (vdW) forces provide an opportunity to combine layers of two or more materials with different properties to engineer new functional heterostructures [2]. These structures are typically engineered to exhibit novel behaviors that are not present in individual components, making them highly useful for advanced applications in biosensors, electronics, photodetectors, solar cells, and energy storage [3,4,5,6,7,8,9,10]. By engineering the interactions between these layers, heterostructures can provide enhanced control over electronic band structures, charge carrier mobility, gate tunability, and optical responses [11,12,13].

**Figure 1:**
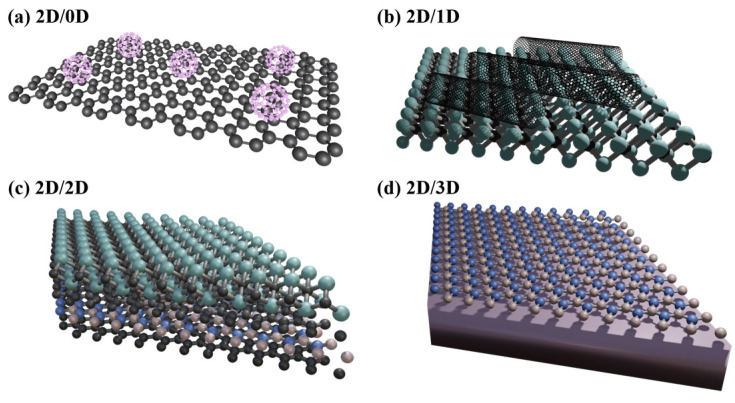
Van der Waals heterostructures. (**a**) 0D nanoparticles or QDs, (**b**) 1D nanowires, (**c**) 2D nanosheets, and (**d**) 3D bulk materials.

Several types of heterostructures exist, depending on the dimensionality of the components involved (Figure 1). Various materials can be used to synthesize heterostructures, based on their target applications and desired properties. Graphene, MoS_2_, WS_2_, WSe_2_, h-BN, and black phosphorus are widely employed in 2D heterostructures owing to their unique electronic and optical properties [14,15,16,17,18]. Moreover, transition metal carbides, nitrides, and carbonitrides (MXenes), new emerging 2D materials, have also been utilized in heterostructures to make use of their advantageous characteristics [19,20,21,22]. These materials can be stacked or interfaced with other 2D layers or three-dimensional (3D) materials to form heterostructures with tunable bandgaps and enhanced charge-transport properties. Additionally, conventional semiconductors, which are 3D materials, including silicon, gallium nitride (GaN), and gallium arsenide (GaAs), are commonly integrated with 2D materials to enhance their optoelectronic performance, particularly in applications such as light-emitting diodes (LEDs), perovskite solar cells, and high-speed transistors [23,24,25]. Zero-dimensional (0D) materials, such as quantum dots (QDs), can be combined with 2D materials to enhance the optical and electronic characteristics of devices [26,27]. One-dimensional (1D) materials such as carbon nanotubes (CNTs) and semiconductor nanowires (e.g., silicon and indium phosphide) can also be integrated with 2D materials to create high-mobility transistors, devices, or sensors [28,29,30].

Bandgap engineering in materials involves modifying electronic properties, particularly the bandgap, to optimize them for specific applications such as transistors, sensors, and optoelectronic devices [31,32,33]. The construction of heterostructures requires that the energy bands of the two semiconductors align with overlapping conduction and valence bands. Therefore, when designing new materials with improved absorption and transport properties, it is crucial to accurately describe the bandgap and the positions of states at the band edges [34]. In particular, bandgap engineering serves as an effective approach to addressing the limitations of inherent material properties and designing advanced material properties and functionalities. For more precise bandgap engineering, the choice of exchange-correlation function is also important. Mori-Sánchez et al. demonstrated that errors in the energy of fractional charges in finite systems propagate to larger systems, leading to systematic inaccuracies [35]. They emphasized that the localization size, defined as the spatial extent of the electron wavefunction, plays a crucial role in predicting accurate bandgaps, underscoring the importance of addressing these issues in density functional theory (DFT) calculations. 

Utilizing DFT for bandgap engineering and band structure prediction is a cutting-edge computational strategy. DFT offers robust insights into electronic properties, significantly enhancing the understanding and design of heterostructures. This review provides a comprehensive integration of bandgap engineering and the prediction of advanced heterostructures by using DFT, and its application. Furthermore, it addresses key limitations and challenges in this rapidly evolving field and proposes solutions to overcome these obstacles.

## 2. Bandgap Engineering in 2D Heterostructures

### 2.1. Methods of Bandgap Engineering in Heterostructures via DFT

Bandgap engineering has become an essential approach for designing materials for specific applications. In heterostructures, the bandgap can be tailored through various mechanisms, enabling the tuning of electronic and optical properties. Researchers have manipulated the bandgap experimentally without using DFT. Chen et al. [36] tuned conduction band offsets by using different aluminum compositions during the synthesis of GaAs/Al_x_Ga_1-x_As nanowires to manipulate the bandgap. This structural modification allows for the optimization of absorption and emission characteristics, which is essential for photovoltaics and light-emitting devices. Cingolani et al. [37] investigated Zn_1−x_Cd_x_Se/ZnSe multiple-quantum-well (MQW) heterostructures, focusing on their electronic and optical properties. Their study provided insights into excitonic transitions, bandgap engineering, and strain effects, showcasing the potential of these heterostructures for optoelectronic applications in the blue and blue-green spectral regions. Fernández et al. [38] conducted a comprehensive study on bandgap structure engineering of ZnS/GaAs heterostructures grown using low-pressure Metal–Organic Vapor Phase Epitaxy (MOVPE). Their research highlighted MOVPE’s capability to produce high-quality ZnS layers with precise control over optical and electronic properties. The study emphasized the material’s sharp excitonic features, strain management, and temperature-dependent bandgap behavior, showcasing its potential for advanced optoelectronic applications such as ultraviolet (UV) and blue-light-emitting devices. Sehar et al. [39] tuned the bandgap of Bi_2_MoO_6_ using transition metals such as Cu^2+^ and Ni^2+^ ions. They revealed that the hybridization of Cu and Ni d-orbitals with the Bi_2_MoO_6_ electronic structure alters the density of states near the band edges. Therefore, this shifts the absorption edge to longer wavelengths, resulting in a narrowing bandgap. Furthermore, they demonstrated that doping creates oxygen vacancies and defects that alter electric transitions. Oxygen vacancies act as electron traps, reducing the recombination of electron–hole pairs, thereby enhancing light absorption and photocatalytic activity.

**Figure 2:**
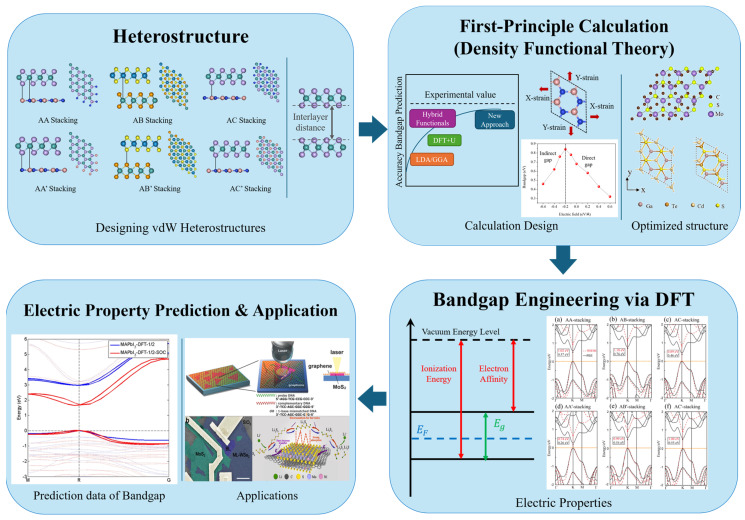
Procedure for the bandgap engineering of heterostructures via DFT. Reprinted with permission from [48], Copyright 2017, Springer Nature. Reprinted with permission from Loan et al. [3], Copyright 2014, Wiley-CH Verlag GmbH & Co. KGaA. Adapted with permission from [49], Copyright 2014, American Chemical Society. Adapted from [40], Copyright 2022, with permission from Elsevier. Adapted from [42], Copyright 2020, with permission from Elsevier. Adapted from [50], Copyright 2021, with permission from Elsevier.

Recently, they have implemented DFT to predict the band structure of materials. This predictive capability supports the synthesis of new materials and the design of novel materials for targeted applications. As shown in Figure 2, the steps for engineering the bandgap of a heterostructure are as follows: (1) designing the heterostructure, (2) designing first-principle calculation methods, (3) performing bandgap engineering via DFT, and (4) predicting the electrical properties and applications. Heterostructures are designed based on their intended applications. A common approach involves combining 2D materials with other 2D materials, although other configurations such as 2D/0D, 2D/1D, and 2D/3D are also possible. Additionally, the stacking method affects the band structure, and selecting an appropriate interlayer distance is essential. The electronic properties of heterostructures vary depending on the design. Meng et al. investigated the electronic properties of GeC/MoS_2_ heterostructures based on different stacking configurations [40]. Their findings indicated that an AB-stacked GeC/MoS_2_ heterostructure, the most stable configuration, exhibited type-II band alignment with an intrinsic direct bandgap, enabling the efficient separation of photogenerated electron−hole pairs. 

The second step is to design first-principles calculation conditions such as exchange-correlation (XC) functionals, electric fields, and strain, which are the most critical steps for predicting and engineering the band structure of heterostructures. Kang et al. reported that the band structure of multilayer 2H-phase TMDs, such as WS_2_, MoS_2_, WSe, and MoTe_2_, is highly responsive to various physical parameters, including thickness, stacking, strain, and electric field [41]. They explained that although variations in thickness and stacking affect the overall bandgap magnitude, they have a minimal impact on the local gap at the K point, which plays a crucial role in the optical properties of 2H-TMDs. Jia et al. demonstrated that applying an external electric field can significantly alter the band structure of the GaTe/CdS hetero-bilayer, inducing transitions from an indirect to a direct bandgap and from a semiconductor to a metal under a critical electric field [42]. Additionally, biaxial strain plays a crucial role in tuning the bandgap, leading to transitions from a direct to an indirect bandgap and from a semiconductor to a metal within the elastic range of the material. Huang et al. investigated the bandgap engineering of an h-BN/MoS_2_/h-BN heterostructure by applying an external electric field [43]. They revealed that the bandgap of the h-BN/MoS_2_/h-BN heterostructure transitioned from indirect to direct as the electric field increased. After designing the heterostructure and first-principles calculation conditions, the bandgap energy of the heterostructure was calculated to predict and analyze the band structure based on the established conditions and design. This aspect will be discussed in more detail in Section 2.3, “Case Studies of Bandgap Engineering in 2D Heterostructures Via DFT”.

DFT calculations allow for quantitative analysis of the influence of various physical parameters on the band structure, providing crucial insights for optimizing the electronic and optical properties. Ultimately, this process is essential for designing ideal materials for application in nanoelectronics and optoelectronics. In particular, engineering the proper band structure and predicting the electrical properties of these types of heterostructures are crucial in this industry. Wi et al. introduced a plasma-assisted doping method to substantially enhance the photovoltaic performance of multilayer MoS_2_ [44]. Jameel et al. investigated the tuning of the bandgap and optical properties of TMDs, including WS_2_ and MoS_2_, using GGA-PBE approximations [45]. Their results showed that MoS_2_ and MoSe_2_ demonstrate higher optical conductivity, reflectivity, and improved optical characteristics owing to their reduced bandgap values. This study highlights the strong potential of the TMD family for photocatalytic and solar cell applications through bandgap engineering. Liu et al. discovered, through DFT calculations and experimental studies of electronic and optical properties, that the continuous incorporation of [MO_4_]^4^⁻ tetrahedral (M = Si, Ge, Sn, and Mn) into the crystal lattice can substantially reduce the bandgap [46]. They demonstrated that bandgap engineering could overcome the limitations of apatite-based compounds for applications in catalysis and energy devices. Krivosheeva et al. conducted a theoretical study using DFT to assess the impact of doping Te atoms by substituting chalcogen atoms in the WS_2_/WSe_2_ heterostructure on electronic properties [47]. They demonstrated that bandgap tuning could facilitate the creation of multilayer structures with adjustable bandgaps, which is crucial for the development of the next generation of electronic and photonic devices.

### 2.2. Case Studies of Bandgap Engineering in 2D Heterostructures via DFT

In this section, studies on the design of graphene, TMD, and carbon nitride-based heterostructures and their corresponding bandgap modulation using DFT calculations are reviewed. Many studies focus on the changes in the bandgap upon the formation of heterostructures. Furthermore, some of these investigations explore modifications in the bandgap induced by external factors, such as electric fields and strain, as well as the impact of structural defects. Applying strain and electric fields to heterostructures modifies the electronic structure of materials by altering their symmetry and causing changes in the energy levels of electrons and holes, respectively [51,52,53]. Structural defects in materials introduce localized states and create trap levels within the bandgap, leading to modifications in the band structure [54,55]. These effects are often utilized to enhance the performance of optoelectronic devices by modulating their electronic structures.

#### 2.2.1. Graphene-Based Heterostructure

The inherent unique features of graphene have made it possible to adopt it for various applications, such as electronic and photonic devices, with high efficiency [56,57,58]. Its exceptional characteristics allow it to support rapid electron transport, durable device structures, effective heat dissipation, and applications in transparent electronics such as touch screens and solar cells [59,60,61,62,63,64]. Despite its many advantages, the absence of a bandgap limits graphene’s effectiveness in specific applications such as transistors, sensors, and optoelectronic, highlighting the need for band structure modulation to achieve further advancements in this field. Research on graphene-based heterostructures has been underway as a means to overcome this limitation.

Kong et al. investigated the modification of the bandgap after combing graphene with Ti_3_C_2_O_2_ MXene QDs to explore its electrocatalytic performance in the hydrogen evolution reaction (HER) [65]. The research reveals that weak vdW interactions and polarization between graphene and Ti_3_C_2_O_2_ QDs in the heterostructure induce a bandgap opening, transforming graphene’s original zero bandgap to 0.39 eV and altering its electronic properties. The interaction influences electronic states, leading to electron accumulation and disrupting the π-bonding balance in graphene, which significantly alters its electronic characteristics. Furthermore, the C–O interactions in the QDs lead to unstable configurations that drive QD aggregation due to antibonding states, while the distortion of graphene in the heterostructure stabilizes the system by enhancing charge transfer and modifying the electronic structure. Moreover, the d-band center shifts to a lower energy level due to hybridization with graphene, improving hydrogen adsorption and boosting electrocatalytic performance for HER.

**Figure 3:**
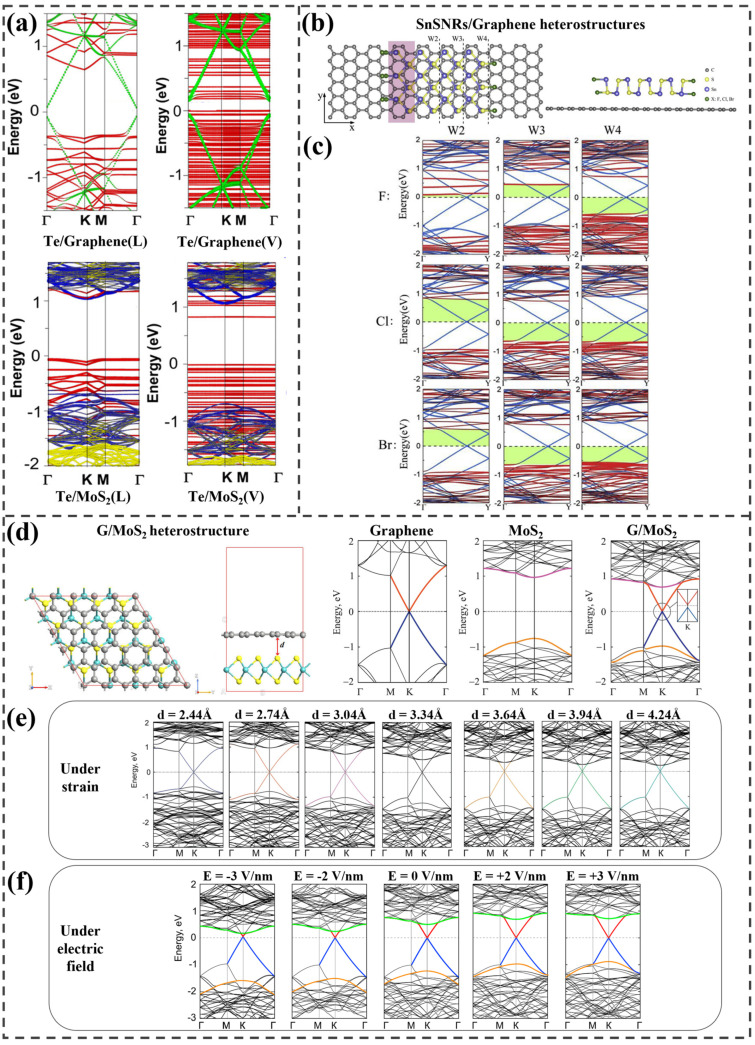
(**a**) Band structure of Te−NR on graphene and MoS_2_ in both lateral (L) and vertical (V) configurations. Te, graphene, Mo, and S are represented in the fatband as red, green, blue, and yellow, respectively Adapted with permission from [66]. Copyright 2022 American Chemical Society. (**b**) Top and side views of the SnSNR/graphene heterostructure, and (**c**) band structures of F−, Cl−, and Br-passivated heterostructures. Adapted from [67], Copyright 2018, with permission from Elsevier. (**d**) Optimized G/MoS_2_ heterostructure and the electronic structures of monolayer graphene, monolayer MoS_2_, and the G/MoS_2_ heterostructure. The calculated band structures of G/MoS_2_ under (**e**) strain and (**f**) electric field. Adapted from [69], Copyright 2018, with permission from Elsevier. Adapted from [70], Copyright 2018, with permission from Elsevier.

Maji et al. conducted DFT calculations to investigate the hybridization of 1D tellurene nanorods (Te-NRs) with 2D graphene and MoS_2_ monolayers for applications in broadband photonics and energy harvesting [66]. The study explored four types of 1D/2D heterostructures: Te-NRs on graphene and MoS_2_ in both lateral (L) and vertical (V) configurations. To evaluate the contributions of structural distortion, interlayer charge redistribution, and quantum confinement, they analyzed the decoupled band structures of each component in the four optimized heterojunctions. The calculated band structures are shown in Figure 3a. In the lateral Te/graphene heterojunction, the carrier transfer from Te to graphene opens a direct bandgap of 130 meV, forming a type-I heterojunction. The vertical Te/graphene heterojunction shows quantum confinement due to the Te-NRs, introducing mid-gap states in Te, resulting in a 150 meV direct gap with a Z-scheme and type-II alignment, leading to efficient charge separation and n-type doping. In the lateral Te/MoS_2_ heterojunction, a type-II heterojunction is formed, where the valence band maximum (VBM) is mainly contributed by Te-NR and the conduction band minimum (CBM) by MoS_2_, resulting in an indirect bandgap of 1.23 eV in the near-infrared range. Charge transfer from Te to MoS_2_ leads to n-type doping, improving electron−hole separation, and enhancing the structure’s potential for solar energy applications. In the vertical Te/MoS_2_ heterojunction, type-I alignment occurs, with quantum confinement reducing the mid-gap states and enhancing exciton lifetimes and optoelectronic performance through charge transfer. 

The study of SnS nanoribbon (SnSNR)/graphene heterostructures by Xia et al. focused on bandgap engineering and electronic properties, exploring the impact of nanoribbon width, edge passivation, and quantum confinement [67]. As illustrated in Figure 3b, three nanoribbon widths—W2, W3, and W4—were investigated, each passivated by fluorine (F), chlorine (Cl), and bromine (Br) atoms to stabilize the structure. Within the heterostructure, the Dirac cone of graphene aligns near the top of the SnSNR’s valence band, forming a p-type Schottky contact. Increasing the SnSNR width causes a slight decrease in the p-type Schottky barrier height (SBH) due to quantum size effects, which stabilize at larger widths. The electronic properties of the passivated heterostructures were analyzed across different widths, revealing substantial effects on electronic structure and Schottky contact type. The electronic structure of the passivated heterostructures is shown in Figure 3c. In F-passivated structures, the Fermi level approaches the CBM of SnSNR, forming an n-type Schottky contact with narrower widths (0.106 eV for W2, 0.457 eV for W3) but transitioning to p-type at W4 (0.581 eV). In Cl-passivated structures, a transition occurs from n-type (0.786 eV) to p-type as width increases (0.672 eV for W3 and 0.607 eV for W4). Similarly, Br-passivated heterostructures transition from n-type SBH for W2 (0.582 eV) to p-type at W3 (0.652 eV), with a further decrease to 0.565 eV at W4. These passivated structures experience greater changes than the bare heterostructure due to the electronegativity of edge atoms, which attract electrons, as confirmed by differential charge density analysis. These results underscore the influence of SnSNR size and edge passivation on the electronic properties of SnSNR/graphene heterostructures. 

Nguyen et al. investigated how the electronic structure of graphene/MoS_2_ heterostructures is influenced by interface configuration [68], strain [69], and electric field application [70]. Initially, they examined four distinct stacking configurations for various interfaces between graphene and MoS_2_ to identify the most stable arrangement. Figure 3d presents the optimized structure of graphene/MoS_2_, along with the band structures of graphene, MoS_2_, and the combined heterostructure. The graphene/MoS_2_ heterostructure forms an n-type Schottky contact with a band structure that reflects a direct combination of the individual band structures of graphene and MoS_2_. This configuration results in an approximately 4 meV bandgap in graphene, attributed to symmetry breaking due to charge transfer between the two materials. Subsequently, the team examined the electronic properties and Schottky contact characteristics of the heterostructure under varying levels of out-of-plane strain by adjusting the interlayer distance from 2.44 Å to 4.24 Å, with an equilibrium distance of 3.34 Å. This range corresponds to strains between −27% and 27%. The effects of strain on the electronic structure are illustrated in Figure 3e. As the interlayer distance increases, the conduction bands shift toward the Fermi level; however, the Dirac cone remains positioned at the K-point, indicating minimal impact on the electronic structure around the Dirac cone. At equilibrium, the graphene/MoS_2_ interface exhibits an n-type Schottky contact with a barrier height of 0.49 eV. Increasing the interlayer distance preserves the n-type contact, while decreasing it induces a transition from n-type to p-type, indicating that interlayer distance controls both the barrier height and the contact type.

**Figure 4:**
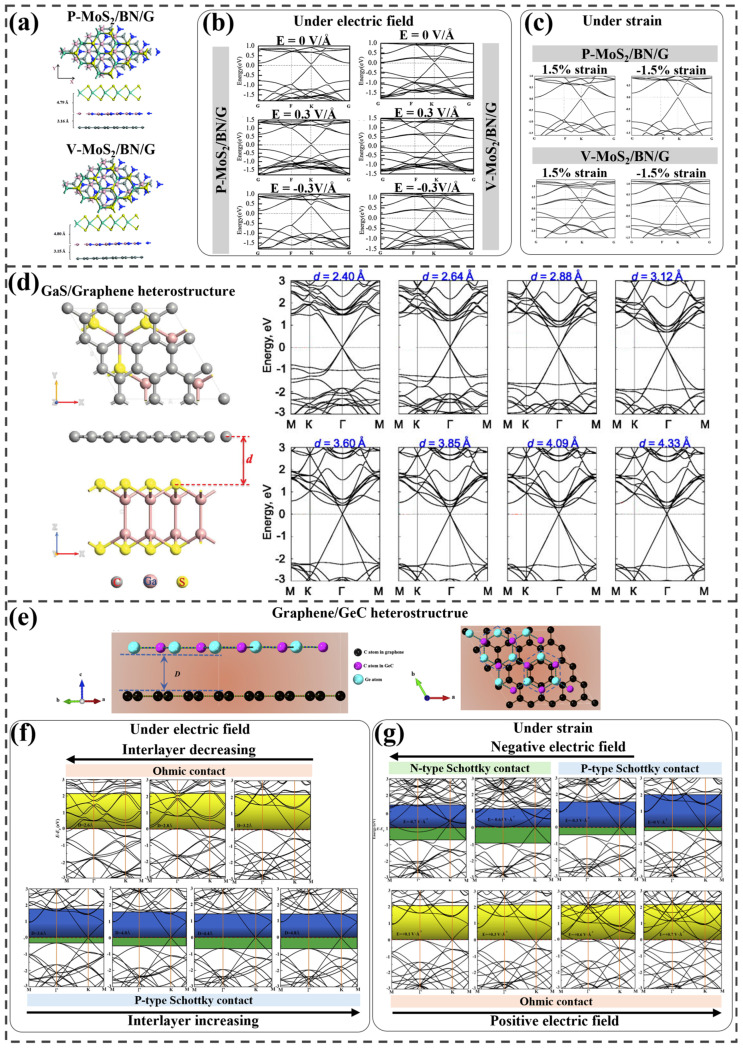
(**a**) Optimized structures of P– and V–MoS_2_/BN/G heterostructures and their electronic structures under (**b**) various electric field strengths and (**c**) strain. Used with permission from the Royal Society of Chemistry, from Ref. [71]; permission conveyed through Copyright Clearance Center, Inc. (**d**) Designed GaS/graphene heterostructure and its band structures under diverse strain conditions. Reprinted from [73], with the permission of AIP Publishing. (**e**) Equilibrium configuration of graphene/GeC and the calculated band structures under varying (**f**) electric field and (**g**) strain. Adapted from [74], Copyright 2019, with permission from Elsevier.

The electronic characteristics of MoS_2_/h-BN/graphene vertical heterostructures were studied under external electric fields and mechanical strain by Zan et al. [71]. Two types of MoS_2_ (pristine and monosulfur vacancies) were used, with interactions mainly governed by vdW forces in the heterostructures, as illustrated in Figure 4a. MoS_2_ gained electrons under negative fields and lost them under positive ones, while graphene showed the opposite behavior. The BN layer consistently lost electrons, and the charge transfer followed the following order: G > P-MoS_2_ > V-MoS_2_ > BN. Without an electric field, P-MoS_2_/BN/G and V-MoS_2_/BN/G have small bandgaps (0.082 and 0.027 eV, respectively). Under a negative field, both became metallic owing to the shift in the Dirac cone of graphene, whereas graphene behaved oppositely (Figure 4b). The SBH increases linearly with a positive electric field. Under a negative field, it initially increases before decreasing, indicating that the graphene layer is effectively doped by shifting its Dirac cone. As shown in Figure 4c, the strain weakened the interlayer coupling but had a minimal effect on the band structures, allowing high carrier mobility to remain stable, even under mechanical deformation.

Sattar et al. [72] conducted bandgap engineering of graphene and boron nitride (GBN) heterostructures, investigating interlayer spacings of 2.4, 2.7, 3.0, 3.3, 3.6, and 4.0 Å. At 2.4 Å, the bandgap opened to 0.87 eV due to interlayer coupling and charge redistribution. As the spacing increased to 4.0 Å, the bandgap gradually decreased to 0.0032 eV, resulting from reduced stress and vdW interactions. Additionally, BN-doped graphene/BN heterostructures—created by replacing the central hexagon in graphene with a BN ring—exhibited a bandgap of 0.49 eV at the Γ symmetry point. These BN-doped heterostructures were designed with six different interlayer distances, paralleling the undoped structures, and their band structures were analyzed. Examination of the bandgap at the K symmetry point of the Brillouin zone showed that increasing the interlayer spacing from 2.4 Å to 4.0 Å produced bandgap values ranging from 1.12 eV to 0.21 eV. This larger bandgap in the BN-doped heterostructure is attributed to the enhanced confinement of charge carriers. As the interlayer distance increased, the stress exerted by the BN layer on graphene decreased, which allowed the charge distribution and sublattice symmetry of graphene to be restored.

The effect of vertical strain on the electronic structure and Schottky contact of a graphene/GaS (G/GaS) heterostructure was explored by Pham et al. Owing to the weak vdW interactions, the band structure of the heterostructure appeared to be a direct combination of the electronic structures of graphene and GaS, forming an n-type Schottky contact with a low barrier height 0.51 eV (Figure 4d) [73]. To study the effect of vertical strain, the interlayer spacing was adjusted from 2.4 to 4.33 Å, with equilibrium at 3.356 Å. For all spacings except 2.4 Å, the n-type SBH remained smaller than the p-type SBH. However, at 2.4 Å, the n-type SBH increased while the p-type SBH decreased, and at 2.60 Å, a transition from n-type to p-type Schottky contact was observed. The modulated band structures under vertical strain reveal a shift in the Fermi level. At 2.4 Å, the Fermi level shifted downward, indicating a transition from n-type to p-type Schottky contact, whereas increasing the interlayer distance from 3.356 to 4.33 Å caused an upward shift in the Fermi level. 

Gao et al. examined the effects of external vertical strains and electric fields on the modulated band structures of a graphene/germanium carbide (g-GeC) heterostructure [74]. The most stable configuration was achieved at an interlayer spacing of 3.413 Å, where a p-type Schottky contact with an SBH of 0.14 eV was observed (Figure 4e). Following the formation of the heterostructure, the GeC bandgap remained approximately 2.10 eV, consistent with the bandgap of the GeC monolayer, while the Fermi level shifted slightly upward, indicating electron transfer from graphene to GeC. To investigate the influence of vertical strain on the electronic properties of the heterostructure, the interlayer spacing was varied. Figure 4f displays the calculated electronic structures for different interlayer spacings. When the spacing was reduced from 3.6 to 2.6 Å, the n-type SBH initially increased before dropping to 2.13 eV, while the p-type SBH nearly diminished to 0. Conversely, increasing the spacing from 3.40 to 4.80 Å resulted in a decrease in the n-type SBH and an increase in the p-type SBH. When the spacing fell below 3.20 Å, the Schottky contact transitioned to an Ohmic contact, with the n-type SBH stabilizing at approximately 2.1 eV. Application of a positive electric field caused a complete transition of the p-type Schottky contact to an Ohmic contact. In contrast, a negative electric field intensified the p-type Schottky contact, leading to an increase in the p-type SBH and a decrease in the n-type SBH, as illustrated in Figure 4g.

**Figure 5:**
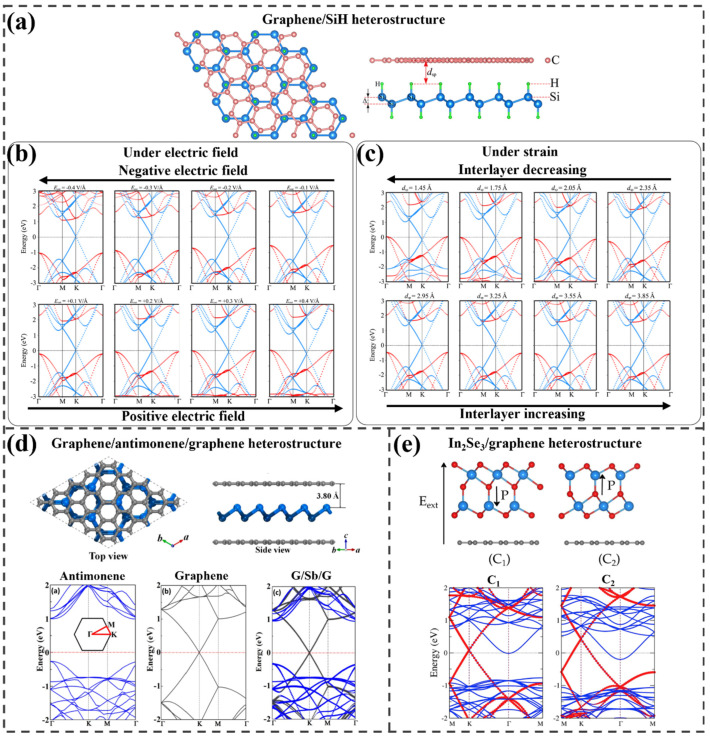
(**a**) Most stable structure of graphene/SiH and its projected electronic structures under diverse conditions of (**b**) electric field and (**c**) strain. Used with permission of the Royal Society of Chemistry, from Ref. [75]; permission conveyed through Copyright Clearance Center, Inc. (**d**) Optimized structure of graphene/antimonene/graphene and the resulting band structures of monolayer antimonene, monolayer graphene, and the heterostructure. Reprinted from [76], Copyright 2019, with permission from Elsevier. (**e**) Relaxed atomic structures of two types of In_2_Se_3_/graphene configurations and their corresponding computed electronic structures. Reprinted from [77], Copyright 2019, with permission from Elsevier.

To investigate band structure engineering, Nguyen et al. developed a graphene/SiH heterostructure (Figure 5a) with a p-type Schottky contact and a barrier of 0.43 eV, which retained the intrinsic properties of both graphene and SiH [75]. Figure 5b illustrates the modifications in the electronic structure under different electric field strengths. When a positive electric field is applied, the n-type SBH increases and the p-type SBH decreases owing to shifts in the SiH band edges, with the VBM approaching the Fermi level and the CBM moving away. At 0.4 V Å⁻^1^, the p-type SBH decreases to zero, transforming the contact into a p-type Ohmic contact. Conversely, under a negative electric field, the p-type SBH rises and the n-type SBH falls, leading to a transition from p-type to n-type Schottky contact at −0.4 V Å⁻^1^. With an increase in the interlayer distance from 1.45 to 3.85 Å, the p-type SBH increases, while the n-type SBH decreases. At 1.45 Å, the p-type SBH reaches zero, converting the contact from Schottky to Ohmic, as shown in Figure 5c. Due to the considerable disparity between the p- and n-type SBHs, modifying the interlayer distance alone is insufficient to induce a p-to-n Schottky contact transition. As the interlayer distance widens, the band edges of the SiH layer shift downward, resulting in an increase in the p-type SBH and a decrease in the n-type SBH.

Su et al. designed a graphene/antimonene/graphene (G/Sb/G) heterostructure to investigate its properties as a potential anode material [76]. The optimized structure and band structure are presented in Figure 5d. An equilibrium G/Sb/G heterostructure is achieved with an interlayer distance of 3.80 Å between graphene and antimonene. The projected band structure of the G/Sb/G heterostructure reveals that the band dispersions of graphene and antimonene are approximately a simple sum of their components, attributed to weak vdW interactions. This interaction preserves the electronic properties of the materials, while the incorporation of graphene enhances conductivity, and the semi-metallic character improves electron transport efficiency. Charge density difference analysis demonstrates that most of the charge accumulates around the antimonene layer, whereas the inner surfaces of the graphene layers exhibit charge depletion. This observation indicates a charge transfer from graphene to antimonene, driven by the vdW forces between the two materials in the G/Sb/G heterostructure.

Ayadi et al. constructed an In22Se33/graphene heterostructure and investigated its modified electronic properties [77]. Due to the asymmetry of the ferroelectric layer α-In22Se33, two distinct configurations (C1 and C2) are possible, both exhibiting typical vdW interactions, as illustrated in Figure 5e. Binding energy calculations indicate that the C2 configuration is approximately 1.80 meV/atom more stable than C1. In the band structure analysis, the Dirac cone of graphene is preserved despite the p-type doping induced by In22Se33. The doping level is influenced by the polarization direction: in configuration C1, the Dirac point and the CBM shift by approximately 0.09 eV and −0.013 eV, whereas in configuration C2, these shifts are more significant, approximately 0.43 eV and −0.21 eV, respectively (Figure 5e). Electron density calculations confirm charge accumulation on In22Se33 and charge depletion on graphene, consistent with the observed p-doping. Notably, configuration C2 demonstrates a larger charge transfer between the layers, resulting in a more pronounced shift of the Dirac point compared to configuration C1.

The theoretical works discussed above on graphene-based heterostructures primarily focus on combining 2D graphene with materials ranging from 0D to 2D. While there are papers reporting bandgap opening and others where it does not occur, all these studies demonstrate van der Waals (vdW) bonded heterostructures. Even in cases where bandgap opening happens, the resulting gap is generally small, as the intrinsic electronic properties of graphene are largely preserved. These studies aim either to mitigate the drawbacks of graphene’s zero bandgap in optoelectronic devices by improving ultrafast recombination, or to leverage graphene’s exceptional characteristics, such as high conductivity, large surface area, and excellent stability, in synergy with other materials in the heterostructures. However, a significant limitation is that most of these calculations utilize the PBE functional, which may lead to an underestimated bandgap, as shown in Table 1. Therefore, further studies utilizing more accurate hybrid functionals are needed to improve the precision of these results.

#### 2.2.2. TMD-Based Heterostructure

The distinctive properties of TMDs, including a tunable bandgap, direct bandgap in monolayer form, high carrier mobility, and strong light–matter interactions, have enabled their use in high-performance electronic, photonic, and sensing devices [78,79,80,81]. Despite these strengths, TMDs still face limitations, such as their sensitivity to environmental degradation, which can impact device performance and longevity [82,83]. Additionally, while TMDs exhibit a direct bandgap in monolayer form, they transition to an indirect bandgap in multilayered structures, limiting their efficiency in certain optoelectronic applications [80]. To address these challenges and further enhance TMDs’ applicability, research is actively exploring TMD-based heterostructures and band structure engineering techniques, aiming to achieve improved electronic, optical, and mechanical properties [84,85]. 

An h-BN/MoS_2_/h-BN sandwich heterostructure was constructed by Huang et al. to investigate its structural and electronic properties, with a focus on bandgap modulation under applied electric fields to explore the potential for tuning the bandgap of monolayer MoS_2_ confined between h-BN layers [43]. In the absence of an external field, the heterostructure exhibits an indirect bandgap, with the CBM at the K point and the VBM at the Γ point, both within the MoS_2_ monolayer. When an external electric field of 0.1 V/Å is applied, the indirect bandgap transitions to a direct bandgap, with the CBM and VBM both shifting to the K point. As the electric field increases beyond 0.1 V/Å, the structure maintains its direct bandgap character, though the bandgap decreases significantly, reaching 0.44 eV at 0.5 V/Å. These findings demonstrate that the electronic band structure of the h-BN/MoS_2_/h-BN heterostructure can be efficiently modulated by an electric field.

**Figure 6:**
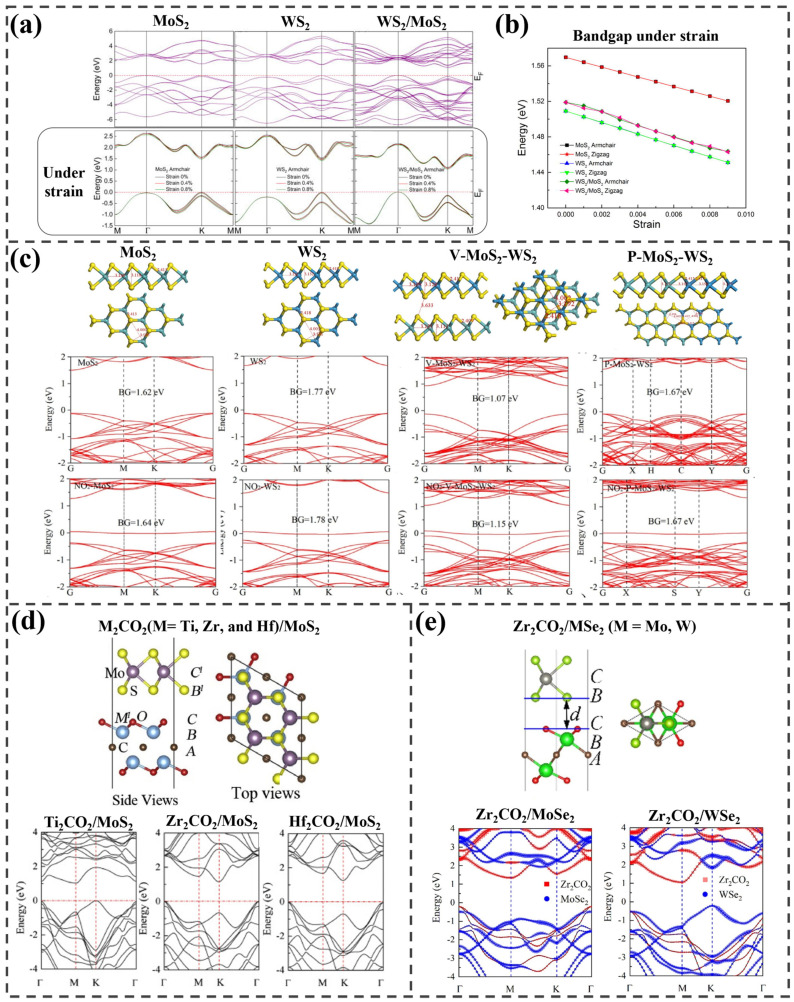
(**a**) Computed band structures with and without strain for single−layer MoS_2_, single−layer WS_2_, and WS_2_/MoS_2_, and (**b**) a plot showing bandgap as a function of strain for these materials. Reprinted from [15], with the permission of AIP Publishing. (**c**) Relaxed structures of monolayer MoS_2_ and WS_2_, as well as heterostructures of V− and P/MoS_2_/WS_2_, along with the calculated electronic structures of the corresponding designed structures with and without NO_2_. Adapted from [86], Copyright 2022, with permission from Elsevier. (**d**) Most stable heterostructure consisting of M_2_CO_2_ (M = Ti, Zr, and Hf) and MoS_2_ and the corresponding computed band structures. Adapted from [88], Copyright 2020, with permission from Elsevier. (**e**) Optimized structure of Zr_2_CO_2_/MSe_2_ (M = Mo and W) and the computed projected band structures of both materials. Adapted from [89], Copyright 2022, with permission from Elsevier.

He et al. explored bandgap modulation through strain engineering for monolayer WS_2_, MoS_2_, and an MoS_2_/WS_2_ heterostructure using both experimental and theoretical approaches [15]. The study revealed that both MoS_2_ and WS_2_ monolayers have direct bandgaps, while the MoS_2_/WS_2_ heterostructure exhibits an indirect bandgap (Figure 6a). The calculations revealed a consistent, linear decrease in the bandgap with strain ranging from 0 to 0.9%, regardless of whether the strain was applied along the zigzag or armchair directions, as shown in Figure 6b. Experimentally, photoluminescence (PL) spectra were obtained for the three materials, with and without strain up to 0.64%, and redshifted peaks indicated changes in the optical bandgap, matching the theoretical results.

The electronic structures of MoS_2_, WS_2_, and the MoS_2_-WS_2_ heterostructure were calculated by Tang et al. to explore the NO_2_ sensing mechanism [86]. In Figure 6c, vertical and lateral heterostructures (V- and P-MoS_2_-WS_2_) were established with bandgaps of 1.07 eV and 1.67 eV, respectively. In the MoS_2_/WS_2_ heterojunction, electrons transfer from metal atoms to non-metals, stabilizing the system’s electronic state, as confirmed by charge analysis. Five gasses were absorbed on the V-MoS_2_-WS_2_ structure, with NO_2_ showing the best adsorption effect and the most stable state when absorbed on the S atoms of WS_2_, while also exhibiting the lowest adsorption energy. When calculating NO_2_ adsorption on the P-MoS_2_-WS_2_, MoS_2_, and WS_2_ structures, the adsorption energy in the P-MoS_2_-WS_2_ structure was found to be the same as that in the V-MoS_2_-WS_2_ structure. Therefore, the band structure was calculated to determine the more favorable configuration. As a result, the V-MoS_2_-WS_2_ structure with adsorbed NO_2_ showed the largest bandgap change, indicating the most significant shift in conductivity after NO_2_ adsorption. This suggests that V-MoS_2_-WS_2_ is more sensitive to NO_2_ compared to other structures.

Defected TMDs combined with graphene heterostructures were constructed by Chen et al. to investigate the electronic structure and bandgap, aiming to explore the catalytic activity for HER [87]. Four heterostructures (MoS_2_, WS_2_, MoSe_2_, and WSe_2_) were prepared, and three types of defected MX_2_/graphene heterostructures were created: (i) MX_2_/G_V_M_ with a missing Mo or W atom, (ii) MX_2_/G_V_X_ with a missing S or Se atom, and (iii) MX_2_/G__V(M+X)_ with both a Mo(W) atom and its connected S(Se) atom removed. First, MX_2_/G_V_X_s structures were confirmed to display the best HER performance by calculating the Gibbs free energies of H-adsorbed systems with and without defects. In pure MX_2_/graphene systems, the semiconducting properties of MX_2_ and the metallic nature of graphene combine, resulting in a slight reduction in the MX_2_ bandgap and the opening of a small gap in graphene, which enhances hydrogen adsorption compared to free-standing MX_2_. Additionally, introducing X or M vacancies forms impurity states near the Fermi level, enhancing MX_2_ conductivity and creating vacant states for hydrogen adsorption, which boosts catalytic performance. In summary, atomic defects activate nearby sites, shift the band center toward the Fermi level, increase adsorption capacity, and lower Gibbs free energy.

Xu et al. studied the electronic structure of three different types of M_2_CO_2_ (M = Ti, Zr, and Hf)/MoS_2_ bilayer heterostructures constructed with the most stable B’C’ stacking [88]. The Ti-containing heterostructure has a bandgap of 0.94 eV between the K and M points, whereas the Zr- and Hf-containing systems exhibit bandgaps of 1.15 and 1.28 eV between the K and Γ points (Figure 6d), respectively. The findings indicate that the integration of M_2_CO_2_ with MoS_2_ significantly affects the band structure compared to the individual systems. The bandgaps of all the heterostructures are smaller than those of MoS_2_ and M_2_CO_2_ monolayers, with an indirect gap. The VBM and CBM in the Ti-containing heterostructure are contributed by the MoS_2_ and Ti_2_CO_2_ layers, respectively. In contrast, for the other two bilayers, the VBM is primarily influenced by M_2_CO_2_ and the CBM by MoS_2_ layers.

The Zr_2_CO_2_ MXene forms vdW bilayer heterostructures with MoSe_2_ and WSe_2_, as reported by Xu et al. [89], both with and without a twist angle. The most stable BC-stacking structure for Zr_2_CO_2_/MoSe_2_ and Zr_2_CO_2_/WSe_2_ was identified, followed by the construction of twisted bilayers with six rotation angles from 13.2° to 60°. In the twisted structures, increased rotation angles led to higher binding energy and interlayer distance, indicating reduced stability. Nearly flat bands near the Fermi level were found in the 46.8° twisted Zr_2_CO_2_/WSe_2_ system but not in the Zr_2_CO_2_/MoSe_2_ structure. Additionally, the HSE06 functional was used to confirm accurate band edges in the designed heterostructures. As shown in Figure 6e, both Zr_2_CO_2_/MoSe_2_ and Zr_2_CO_2_/WSe_2_ are indirect semiconductors, with bandgaps of 1.55 eV and 1.44 eV, respectively. In Zr_2_CO_2_/MoSe_2_, the VBM is at the Γ point and the CBM at the M point, while in Zr_2_CO_2_/WSe_2_, the band edges are at the K and M points. Zr_2_CO_2_/MoSe_2_ forms a type-I heterojunction, with conduction and valence bands primarily derived from Zr_2_CO_2_. In contrast, Zr_2_CO_2_/WSe_2_ forms a type-II heterojunction, with WSe_2_ dominating the VBM and Zr_2_CO_2_ the CBM, allowing effective separation of photoexcited electrons and holes.

Sung et al. investigated the impact of the interfacial structure between 2D-MoS_2_ and 3D-GaN by examining its electronic properties, focusing on the interaction between MoS_2_ and two surface terminations of GaN (Ga-GaN and N-GaN) [90]. To explore the interface behavior, both monolayer (1L) and bilayer (2L) MoS_2_ were analyzed on Ga- and N-terminated GaN surfaces, providing insight into how these configurations affect the electronic structure. For the MoS_2_/Ga-GaN and MoS_2_/N-GaN heterostructures, according to structure analysis, MoS_2_ bonds with the Ga-GaN surface, eliminating surface states, while on N-GaN, hybridization with MoS_2_ leads to metallic behavior. Spin-splitting occurs in both structures due to GaN surface polarization, even without an external electric field. Hybridization influences electronic conduction, as shown by PDOS and partial charge density calculations, particularly in the MoS_2_/N-GaN heterostructure. The electronic properties of 2L-MoS_2_ on GaN surfaces vary based on GaN surface termination. Band structure analysis shows BL-MoS_2_ acts as a buffer layer, with TL-MoS_2_ exhibiting n-type behavior on Ga-GaN and p-type behavior on N-GaN. GaN surface termination effectively controls the electrical characteristics of TL-MoS_2_, while energy splitting occurs in BL-MoS_2_, similar to 1L-MoS_2_. This highlights the influence of GaN surface termination on the electronic properties of multilayer MoS_2_/GaN heterostructures.

**Figure 7:**
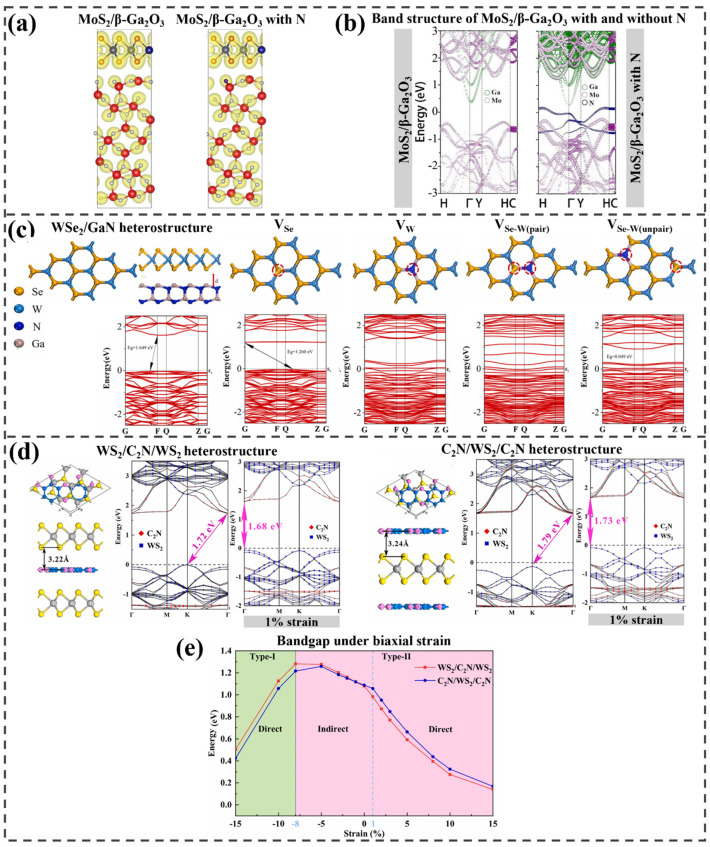
(**a**) Optimized MoS_2_/β−Ga_2_O_3_ heterostructures and their charge density with and without N, and (**b**) the calculated band structures. Reprinted with permission from [91], Copyright 2019, Springer Nature. (**c**) Relaxed atomic structures and obtained electronic structures of WSe_2_/GaN with and without four different types of vacancies. Reprinted from [92], Copyright 2024, with permission from Elsevier. (**d**) Most stable structures of two different stacking configurations consisting of WS_2_ and C_2_N, and their band structures with and without 1% strain. (**e**) Graph representing the transition of bandgap and heterojunction types under different strain strengths. Adapted from [93], Copyright 2022, with permission from Elsevier.

Huan et al. investigated the band alignment in 2D-MoS_2_/3D-β-Ga_2_O_3_ heterojunctions, with a focus on the electronic changes occurring before and after nitridation treatment [91]. Figure 7a illustrates the constructed heterostructures. Through X-ray photoelectron spectroscopy (XPS) analysis and DFT calculations, they explored the behavior of these heterojunctions and their potential impact on nanoelectronic devices. XPS analysis initially showed that the bandgap and band diagram remained unchanged for MoS_2_/β-Ga_2_O_3_ heterojunctions, both with and without nitridation treatment. When calculating the electronic structures of the two heterojunction systems using the DFT method, mid-gap states appeared in the nitrided MoS_2_/β-Ga_2_O_3_ heterojunctions, as shown in Figure 7b. These mid-gap states facilitated charge transfer across the interface, with the resulting interface dipole contributing to the binding energy shift observed in experimental measurements. Additionally, the calculated conduction band offsets were 0.82 eV for the unnitrided and 1.0 eV for the nitrided heterojunctions, closely matching the experimental results. These findings demonstrate that surface nitridation can adjust band offsets.

The impact of various defects on the electronic properties of a WSe_2_/GaN heterostructure, created by placing a 2D WSe_2_ layer on a 3D GaN layer with a consistent interlayer distance of 3.5 Å, was analyzed by Ye et al. [92]; four defect types were studied: VSe (single Se vacancy), VW (single W vacancy), VSe-W(pair) (neighboring vacancies), and VSe-W(unpair) (separate vacancies). The designed heterostructures and their electronic structures are presented in Figure 7c. The pristine heterostructure has an indirect bandgap of 1.649 eV. Introducing an Se vacancy (VSe) reduces the bandgap to 1.260 eV, while the VSe-W(unpair) defect sharply lowers it to 0.049 eV due to impurity levels. In contrast, the band structure with VW and VSe-W(pair) defects differs significantly from those with VSe and VSe-W (unpair) defects. In these cases, impurity levels from vacancy defects cross the Fermi level, causing metallic properties, likely due to vacancy defects and surface-suspended bonds between layers. Overall, reducing the bandgap in the WSe_2_/GaN heterostructure through vacancy defects tunes its electronic properties and enhances electron transition from the valence to the conduction band. Furthermore, PDOS analysis showed that vacancy defects modulated the electronic properties of the WSe_2_/GaN heterostructure, reducing the work function and facilitating easier electron transfer.

Li et al. fabricated three different heterojunctions composed of C2N and WS2 to study their modified electronic properties as promising photocatalysts: C2N/WS2, WS2/C2N/WS2, and C2N/WS2/C2N [93]. The optimized WS2/C2N structure has an interlayer distance of 3.25 Å and an energy gap of 1.79 eV. The most stable WS2/C2N/WS2 and C2N/WS2/C2N configurations have binding energies of −9.23 eV and −14.75 eV, with interlayer distances of 3.24 Å and 3.22 Å, respectively. As shown in Figure 7d, both trilayers exhibit indirect bandgaps of 1.72 and 1.79 eV, with VBM at the K point and CBM at the *Γ* point, dominated by WS2 and C2N, indicating type-II heterojunctions. To transform the indirect gap into a direct band type, the band structure was examined after applying strain. Both the WS2/C2N/WS2 and C2N/WS2/C2N heterostructures exhibited similar behavior under tensile strain, transitioning to a direct bandgap at low strain levels. Figure 7e shows the changes in bandgap and band structure characteristics under strain. As the strain increased, the bandgap gradually decreased until it reached zero. In contrast, under compressive strain, a different pattern was observed. At a critical strain of −8%, both trilayer heterostructures shifted to type-I heterostructures, and a transition from an indirect to a direct band type occurred. Therefore, by applying strain, it is possible to design heterostructures with three distinct band configurations: a type-II heterostructure with a direct bandgap, a type-II heterostructure with an indirect bandgap, and a type-I heterostructure with a direct bandgap.

In the described TMD-based heterostructure works, TMD materials are combined with various 2D or 3D materials with vdW, as summarized in Table 2, and the electronic structures of these designed heterostructures are evaluated through DFT calculations to investigate alterations in their optical and electronic features. These studies exhibit remarkable bandgap modulation through external factors such as strain, electric fields, and defect engineering. However, it is also necessary to investigate the heterostructures involving several layers of TMDs instead of monolayer TMDs. One of the critical characteristics of TMDs is that they exhibit a direct bandgap in monolayer form, while they have an indirect bandgap in bulk form. This property is a significant consideration for optical and electrical field applications. In practical implementation, the application of monolayer TMDs faces technical challenges, leading to the predominant use of bulk TMDs. Therefore, research and understanding of the electronic structures of heterostructures composed of bulk TMDs are expected to greatly contribute to practical applications.

#### 2.2.3. Carbon Nitride-Based Heterostructures

Carbon nitride’s unique attributes including its remarkable chemical and thermal stability, moderate bandgap, and effectiveness under visible light have made it advantageous for use in photocatalysis, energy storage, and optoelectronic applications [94,95,96]. Its photocatalytic activity, driven by visible light absorption, makes it an ideal candidate for sustainable applications, such as water splitting and CO_2_ reduction [97,98]. Nevertheless, carbon nitride exhibits certain limitations, such as limited light absorption, rapid recombination of charges, low electrical conductivity, and an indirect bandgap, that collectively restrict its efficiency in advanced optoelectronic applications [99,100]. Efforts to address these challenges have centered on engineering carbon nitride-based heterostructures and modifying its band structure, thereby refining its electronic and optical properties to achieve higher efficiencies in photocatalytic and optoelectronic applications [101,102].

**Figure 8:**
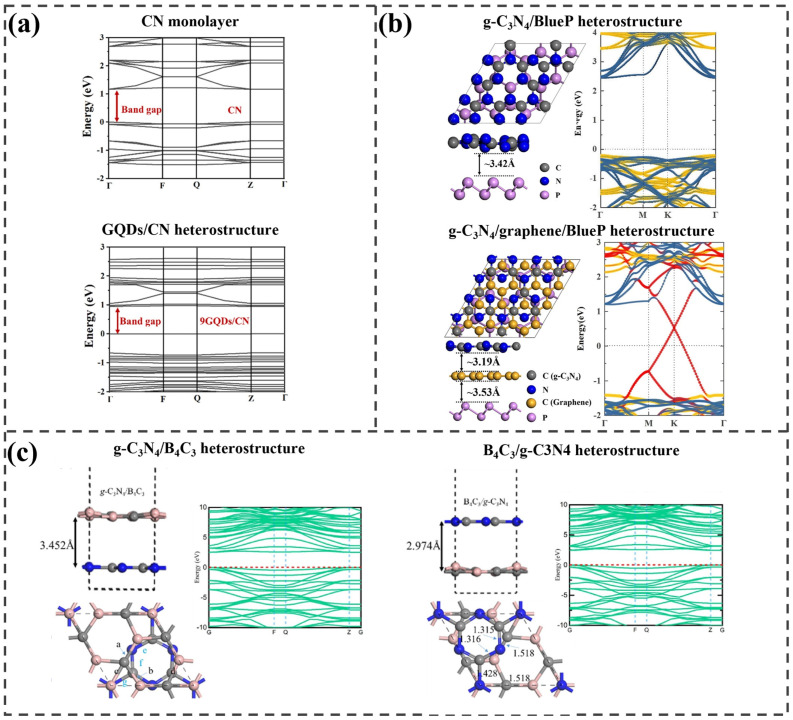
(**a**) Calculated electronic structures of the optimized CN and GQDs/CN structures. Adapted from [103], Copyright 2022, with permission from Elsevier. (**b**) Relaxed atomic structures and their electronic band structures of g−C_3_N_4_/BlueP heterostructures, with and without a graphene monolayer between them. The fatband colors for g−C_3_N_4_, graphene, and BlueP are yellow, red, and blue, respectively. Adapted from [104], Copyright 2024, with permission from Elsevier. (**c**) Most stable atomic structures of two different configurations consisting of g-C_3_N_4_ and B_4_C_3_, and the band structures of the two designed heterostructures. Reprinted from [105], Copyright 2020, with permission from Elsevier.

Graphene quantum dots (GQDs) combined with g-C_3_N_4_ have been introduced as effective photocatalysts for NO removal under visible light, with the design guided by Cui et al. [103]. In this work, DFT calculations were performed for an GQDs/g-C_3_N_4_ heterostructure to clarify the improved photocatalytic performance of g-C_3_N_4_. This study shows that electrons accumulate near GQDs while electron density decreases around g-C_3_N_4_, indicating that GQDs are electrostatically adsorbed onto g-C_3_N_4_. This adsorption creates a built-in electric field from g-C_3_N_4_ to GQDs. When light irradiates the GQDs/g-C_3_N_4_ composite, photogenerated electrons are excited from GQDs and move toward g-C_3_N_4_, countering the built-in electric field. Moreover, this study demonstrates that the bandgap decreases and the VBM shifts upward after the adsorption of GQDs onto g-C_3_N_4_, with the VBM located on the GQDs and the CBM on g-C_3_N_4_ in the heterostructure, as shown in Figure 8a. Therefore, this indicates that electrons excited from GQDs transport to g-C_3_N_4_, resulting in the formation of a type-II heterojunction within the GQDs/g-C_3_N_4_ composite. Additionally, these calculations are consistent with the experimental findings. Thus, this heterostructure demonstrated superior photocatalytic properties compared to g-C_3_N_4_.

Cai et al. constructed two heterostructures composed of graphitic carbon nitride (g-C_3_N_4_), blue phosphorene (BP), and graphene, and evaluated their electronic properties to explore their potential as efficient photocatalysts [104]. The optimal configuration of the g-C_3_N_4_/BP heterostructure was identified with an interlayer spacing of 3.42 Å. The HSE06 function was adopted to calculate the band structures with a direct bandgap of 2.67 eV, indicating enhanced photoexcitation. As displayed in Figure 8b, CBM and VBM are dominated by BP and g-C3N4, respectively, indicating the formation of a Z-scheme heterostructure. In the g-C_3_N_4_/G/BP system, the interlayer distances between g-C_3_N_4_/Graphene and graphene/BP were measured to be 3.19 Å and 3.57 Å, respectively. In Figure 8b, compared to the g-C_3_N_4_/BP system, the band structure of g-C_3_N_4_/G/BP exhibits an increase in the VBM and CBM by 0.07 eV and 0.02 eV, respectively. The Dirac point of graphene is observed at 0.52 eV, which indicates its p-doping nature. These band shifts can be attributed to the transfer of electrons from the intercalated graphene to the adjacent layers. This charge redistribution forms built-in electric fields from graphene toward both g-C_3_N_4_ and BP, differing from the g-C_3_N_4_/BP structure but still beneficial for exciton separation.

To enhance the photocatalytic performance of g-C_3_N_4_, Mahmood et al. used 2D B_4_C_3_ to create heterostructures [105]. The g-C_3_N_4_/B_4_C_3_ structure shows a more stable configuration due to strong binding energy, while B_4_C_3_/g-C_3_N_4_ is thermodynamically more stable. Figure 8c presents the electronic structures of the designed heterostructures. The bandgaps of g-C_3_N_4_/B_3_C_4_ and B_4_C_3_/g-C_3_N_4_ were calculated to be 2.537 and 2.459 eV, respectively, using the HSE06 functional. Therefore, it is expected that g-C_3_N_4_ and B_4_C_3_ composites will demonstrate improved light absorption capabilities compared to the standalone g-C_3_N_4_ monolayer due to the significantly reduced bandgap of g-C_3_N_4_ when compared to its isolated monolayer (3.123 eV). This modification enhances electron transfer from the valence band to the conduction band within the composite structure, potentially leading to a red shift in the optical absorption edge.

Zhan et al. focused on calculating the electronic structure of a g-C_3_N_4_/BP system under various strains [106]. The heterostructure shows a stable configuration with an interlayer distance of 3.02 Å, indicating vdW interaction. The g-C_3_N_4_/BP heterojunction was observed as a type-I heterojunction with a 2.35 eV indirect bandgap. The CBM is at the Gamma point influenced by BP, and the VBM is shared by BP and g-C_3_N_4_ at the M point. Type-I heterojunctions reduce photocatalytic efficiency due to poor charge separation, and the indirect bandgap hinders electronic transitions. To overcome this, strain was applied to convert the indirect bandgap into a direct bandgap, and the band structure was analyzed. When biaxial strain from −10% to 10% was applied, the bandgap peaked at −4% and decreased with more strain. The bandgap decreased from 2.35 to 0.97 eV as the tensile strain increased, with a shift to a direct bandgap at 10% strain. Biaxial compressive strain reduced the bandgap, peaking at 2.65 eV at −4% strain. The CBM remains at the Γ point, while the VBM shifts from M to Γ, transitioning the bandgap from indirect to direct beyond −4%. The type-I to type-II heterojunction transition occurs at −4% or higher strain, with BP contributing to the VBM and g-C_3_N_4_ to the CBM, enabling efficient charge separation.

**Figure 9:**
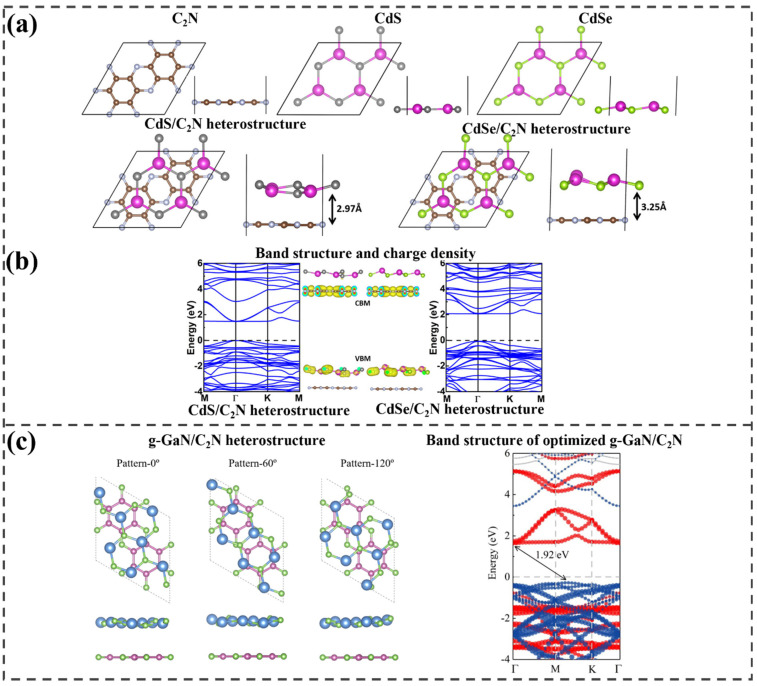
(**a**) Optimized atomic structures of monolayers of C_2_N, CdS, and CdSe, along with the heterostructures of CdS/C_2_N and CdSe/C_2_N; (**b**) computed electronic structures and charge density of the two heterostructures. Adapted with permission from [107]. Copyright 2020 American Chemical Society. (**c**) Relaxed structure of g−GaN/C_2_N with and without rotation and the obtained projected band structure of the most stable heterostructure configuration. The fatband colors for C_2_N and g−GaN are red and blue, respectively. Reprinted from [108], Copyright 2023, with permission from Elsevier.

Kishore et al. constructed CdS/C_2_N and CdSe/C_2_N heterostructures and explored their electronic structures to evaluate their potential as photocatalysts [107]. The optimized interlayer distances were 2.97 Å for C_2_N/CdS and 3.25 Å for C_2_N/CdSe, which are consistent with vdW equilibrium spacings, as illustrated in Figure 9a. In the band structure calculated using the HSE06 functional, the CdS/C_2_N and CdSe/C_2_N heterostructures exhibit direct bandgaps of 1.48 and 2.12 eV, respectively, indicating electronic characteristics favorable for absorbing the solar spectrum. Figure 9b shows that the VBM and CBM at the Γ point for both heterostructures are dominated by the CdS or CdSe layers and C2N, respectively. Consequently, after photoexcitation, the electrons localize in the C_2_N layer, while the holes remain in the CdS and CdSe layers. This spatial separation of charge carriers extends their lifetimes and reduces the recombination rate.

Huang et al. explored the electronic properties of g-GaN/C_2_N heterostructures to examine their photocatalytic characteristics [108]. Three distinct stacking configurations were designed by rotating the g-GaN monolayer by 0°, 60°, and 120° (Figure 9c). In all optimized g-GaN/C_2_N heterostructures, the g-GaN layer shows slight curvature, while the C_2_N layer remains flat. An interlayer distance of approximately 3.5 Å indicates that vdW forces dominate the interface. In the calculation, the initial structure without rotation is identified as the most stable with −30.68 meV/A of binding energy. As presented in Figure 9c, an indirect bandgap of 1.92 eV was obtained in the prepared heterostructure, with the CBM at the Γ point and the VBM positioned between the K and M points. This represents a significant reduction compared to the pristine g-GaN (3.22 eV) and C_2_N (2.48 eV) monolayers. The CBM and VBM originate from separate monolayers, resulting in an offset energy band configuration. The C_2_N monolayer is solely responsible for the CBM, while the VBM comes entirely from the g-GaN monolayer. This offset band arrangement is characteristic of a type-II heterostructure, which aids in the efficient spatial separation of photogenerated charge carriers. Therefore, the g-GaN/C_2_N heterostructure can be classified as a type-II system.

As displayed in Table 3, most of the research was carried out using the HSE06 hybrid functional to investigate the more accurate band structures of the designed heterostructures. This approach is expected to provide more precise insights into the related fields, therefore greatly contributing to the advancement of the respective areas. However, research on carbon nitride is still in early stages compared to graphene and TMD-based heterostructures, suggesting that more studies are required to fully understand its potential and explore its applications in various fields.

### 2.3. Current Limitations and Challenges (Bandgap Engineering in Heterostructures)

One major issue in bandgap engineering is the underestimation of electronic properties by conventional DFT approximations, such as the LDA and GGA, despite their accurate predictions of numerous ground-state properties [109,110]. The weak vdW forces that hold 2D heterostructures together are not adequately described by conventional DFT, which leads to inaccuracies in predicting interlayer distances and energies. These issues arise from the absence of a discontinuity in the exchange-correlation potential when transitioning from the valence band to the conduction band, as well as the presence of a derivative discontinuity in the energy concerning the number of electrons [34,111,112]. Consequently, many researchers propose new computational methods that can predict bandgap energies more accurately than conventional LDA and GGA, particularly for semiconductors and insulators. The choice of XC functionals heavily influences DFT predictions, with varying results depending on the material in question [113]. The rising complexity of heterostructures increases the computational cost of DFT calculations, making comprehensive exploration increasingly challenging. Additionally, strain and defects complicate predictions further, as local distortions may not be accurately captured within conventional DFT frameworks. Ferreira et al. introduced a novel method known as LDA-1/2, which is based on the principles of LDA and half-ionization [114]. The LDA-1/2 method demonstrates improved agreement with experimental results compared to traditional approaches while also providing both accuracy and computational efficiency in calculating the electronic properties of semiconductors. Moreover, hybrid functionals and GW approximations can enhance bandgap predictions, although they come at a higher computational cost. Furthermore, time-dependent DFT (TDDFT) using local XC functionals fails to capture excitonic effects in extended systems, leading to an underestimation of energy values. Given that standard DFT does not account for these interactions, many researchers are exploring advanced methods, such as the GW-Bethe–Salpeter equation (GW-BSE) method, to achieve more accurate predictions [115,116]. Liu et al. employed a first-principles approach that integrates TDDFT with the GW-BSE method to investigate exciton dynamics in 2D vdW heterostructures [117]. This innovative approach facilitates accurate simulations of many-body electron−hole interactions, thereby providing insights into ultrafast charge transfer and excitonic effects that surpass single-particle approximations.

Modeling bilayer heterostructures presents additional challenges due to the formation of moiré patterns, which necessitate large-scale simulations. Furthermore, spin–orbit coupling (SOC) is a crucial factor in materials such as TMDs; however, conventional DFT may overlook this effect. Rossi et al. investigated the influence of SOC on vdW heterostructures, with a particular focus on its impact on electronic properties [118]. Traditional calculations are often conducted at absolute zero temperature, thereby neglecting thermal effects that can significantly influence electronic properties. Zibouche et al. proposed a novel approach to calculate the bandgap energy of TMD bilayers [119]. Their findings indicate that tungsten-based systems close their bandgaps at lower electric fields compared to molybdenum-based systems, a distinction that necessitates consideration of spin–orbit effects. They emphasized that the application of an external electric field induces polarization in the electron density, resulting in anisotropy that substantially enhances SOC. Consequently, the Stark effect intensifies spin splitting in both the valence and conduction bands. Moreover, they demonstrated that spin splitting in centrosymmetric TMD bilayers can occur due to SOC when an external electric field is applied perpendicular to the layers. Addressing these challenges is crucial for accurate bandgap engineering in heterostructures and maximizing their potential applications. Holzer et al. proposed a consistent current density functional theory (CDFT) approach for relativistic DFT, which incorporates SOC [120]. Unlike non-relativistic DFT, SOC leads to a non-zero paramagnetic current density. They emphasized that density functionals dependent on kinetic energy density, such as meta-generalized gradient approximations, should be developed within the CDFT framework. Consequently, they recommend employing the CDFT framework for self-consistent spin–orbit calculations to ensure accuracy.

In conclusion, addressing these limitations and challenges is essential for advancing the practical application of bandgap engineering in heterostructures and realizing their full potential in future technologies.

## 3. Future Prospects and Emerging Trends

The field of bandgap engineering and prediction in 2D material heterostructures is advancing rapidly, presenting significant opportunities for future research and technological breakthroughs. One promising direction involves the development of machine learning (ML) and artificial intelligence techniques that can be integrated with DFT to enhance predictive capabilities and efficiently explore the vast compositional and configurational space of 2D materials [121]. These approaches have the potential to dramatically reduce computational costs while providing more accurate bandgap predictions for novel heterostructures.

Future research must also emphasize experimental validation and scalable fabrication techniques. The combination of advanced DFT simulations with experimental work will ensure that theoretical predictions translate into practical and scalable technologies. Furthermore, the integration of 2D heterostructures into existing semiconductor technologies will be crucial for achieving real-world impact, necessitating research focused on material stability, contact engineering, and device architecture.

Overall, the future of bandgap engineering in 2D heterostructures lies in leveraging interdisciplinary approaches that combine computational methods, novel fabrication techniques, and innovative applications to unlock the full potential of these materials.

## 4. Conclusions

This paper provides a comprehensive analysis of bandgap engineering and band structure prediction in 2D vdW heterostructures utilizing DFT. The study explored the design and engineering of heterostructures, encompassing various stacking configurations, exchange-correlation functionals, and the influence of external parameters, such as electric fields and strain. Furthermore, it not only highlights the current limitations of DFT—such as the underestimation of bandgap energies and the influence of spin–orbit coupling—but also introduces advanced methodologies and emerging trends aimed at overcoming these challenges. The future of bandgap engineering in 2D heterostructures will rely on utilizing integrated approaches, incorporating ML and experimental validation to maximize their potential for next-generation technologies. By addressing these limitations and combining theoretical insights with practical advancements, this research will pave the way for scalable, high-performance applications in electronics, optoelectronics, and energy devices.

## Figures and Tables

**Table 1:** Summary of carbon-based heterostructures, dimensions and functional types used in band structure calculation.

Designed Heterostructure	Dimension	Functional Type for Band Structure Calculation	Reference
Ti_3_C_2_O_2_ QDs/graphene	2D-0D	HSE06	[65]
Te/graphene, Te/MoS_2_	2D-1D	PBE	[66]
SnSNR/graphene	2D-1D	PBE	[67]
Graphene/MoS_2_	2D-2D	PBE	[68]
Graphene/MoS_2_	2D-2D	PBE	[69]
Graphene/MoS_2_	2D-2D	PBE	[70]
MoS_2_/BN/graphene	2D-2D-2D	PBE	[71]
BN-doped graphene/BN	2D-2D	PBE	[72]
Graphene/GaS	2D-2D	PBE	[73]
Graphene/GeC	2D-2D	PBE	[74]
Graphene/SiH	2D-2D	HSE06	[75]
Graphene/Sb/graphene	2D-2D-2D	PBE	[76]
In_2_Se_3_/graphene	2D-2D	PBE	[77]

**Table 2:** Summary of TMD-based heterostructures, dimensions, and functional types used in band structure calculation.

Designed Heterostructure	Dimension	Functional Type for Band Structure Calculation	Reference
h-BN/MoS_2_/h-BN	2D-2D-2D	PBE	[43]
WS_2_/MoS_2_	2D-2D	PBE	[15]
MoS_2_/WS_2_	2D-2D	PBE	[86]
MX_2_/graphene (M = Mo, W; X = S, Se)	2D-2D	PBE	[87]
M_2_CO_2_/MoS_2_ (M = Ti, Zr and Hf)	2D-2D	HSE06	[88]
Zr_2_CO_2_/MSe_2_ (M = Mo, W)	2D-2D	HSE06	[89]
MoS_2_/GaN	2D-3D	PBE	[90]
MoS_2_/β-Ga_2_O_3_	2D-3D	HSE06	[91]
WSe_2_/GaN	2D-3D	PBE	[92]
WS_2_/C_2_N	2D-2D	HSE06	[93]

**Table 3:** Summary of carbon nitride-based heterostructures, dimensions, and functional types used in band structure calculation.

Designed Heterostructure	Dimension	Functional Type for Band Structure Calculation	Reference
GQDs/CN	0D-2D	PBE	[103]
g-C_3_N_4_/graphene/BlueP	2D-2D-2D	HSE06	[104]
B_4_C_3_/g-C_3_N4	2D-2D	HSE06	[105]
g-C_3_N_4_/blue phosphorene	2D-2D	HSE06	[106]
CdX/C_2_N (X = S, Se)	2D-2D	HSE06	[107]
g-GaN/C_2_N	2D-2D	HSE06	[108]
